# Synthetic Biology and Metabolic Engineering for Marine Carotenoids: New Opportunities and Future Prospects

**DOI:** 10.3390/md12094810

**Published:** 2014-09-17

**Authors:** Chonglong Wang, Jung-Hun Kim, Seon-Won Kim

**Affiliations:** Division of Applied Life Science (BK21 Plus), PMBBRC, Gyeongsang National University, Jinju 660-701, Korea; E-Mails: wangchonglong@gmail.com (C.W.); cremoris2000@hotmail.com (J.-H.K.)

**Keywords:** marine carotenoids, carotenoid synthesis, carotenoid modification, metabolic engineering, synthetic biology, protein engineering

## Abstract

Carotenoids are a class of diverse pigments with important biological roles such as light capture and antioxidative activities. Many novel carotenoids have been isolated from marine organisms to date and have shown various utilizations as nutraceuticals and pharmaceuticals. In this review, we summarize the pathways and enzymes of carotenoid synthesis and discuss various modifications of marine carotenoids. The advances in metabolic engineering and synthetic biology for carotenoid production are also reviewed, in hopes that this review will promote the exploration of marine carotenoid for their utilizations.

## 1. Introduction

Carotenoids are a class of naturally occurring pigments originated in the chloroplasts and chromoplasts of plants, algae and some photosynthetic microorganisms [1,2,3,4]. As of 2004, over 750 known carotenoids, which can be divided into xanthophylls (containing oxygen) and carotenes (pure hydrocarbons), have been isolated from natural sources [5]. These structurally diverse pigments play important biological roles in light capture, protection of cells from the damaging effects of free radicals, and synthesis of many hormones as a precursor [6,7,8,9,10]. Carotenoids are traditionally used as food colorants, animal feed supplements, and, very recently, as nutraceuticals and pharmaceuticals [11,12]. Over the past few decades, researches have supported that the ability of carotenoids to reduce the risk of certain cancers, cardiovascular diseases, and degenerative pathogenesis (e.g., Alzheimer and Parkinson) due to their antioxidative properties [13,14]. According to “Carotenoids: A Global Strategic Business Report” from Global Industry Analysts (GIA), the global market for carotenoids was estimated at approximately $1.07 billion in 2010 and is projected to top $1.2 billion by 2015 [15]. Therefore, many efforts have been made to improve the production of these natural compounds for ever-increasing demands [12,16,17].

The ocean is a complex aquatic ecosystem covering about 71% of the Earth’s surface, which is around 300 times larger than the habitable volume of the terrestrial habitats on Earth. A large proportion of all life on Earth lives in the ocean. Ecologically distinct from the terrestrial ecosystem, the ocean constitutes a unique reservoir of marine biodiversity and provides a vast resource of foodstuffs, medicines, and other useful materials. As such, more than 250 novel carotenoids have originated from marine species [10], many of which show great potential in commercial applications [18]. With the advent of synthetic biology and metabolic engineering, many engineering tools including vectors, genetic controllers, and enzyme designing, have been developed for heterologous production of valuable chemicals. These tools create new opportunities for exploring marine carotenoids for food and health industries. In this review, we describe diverse and novel carotenoids from marine resources and summarize recent progresses in synthetic biology and metabolic engineering which provide great application potential for marine carotenoids.

## 2. Diversity of Marine Carotenoids

Many carotenoids have been reported from a wide range of marine species. The advances in current technologies facilitate the elucidation of the carotenoid biosynthetic pathways and relevant enzymes from marine species, which would enable the production of important carotenoids from marine organisms.

### 2.1. Pathways and Diverse Enzymes for Biosynthesis of Carotenoids

**Figure 1:**
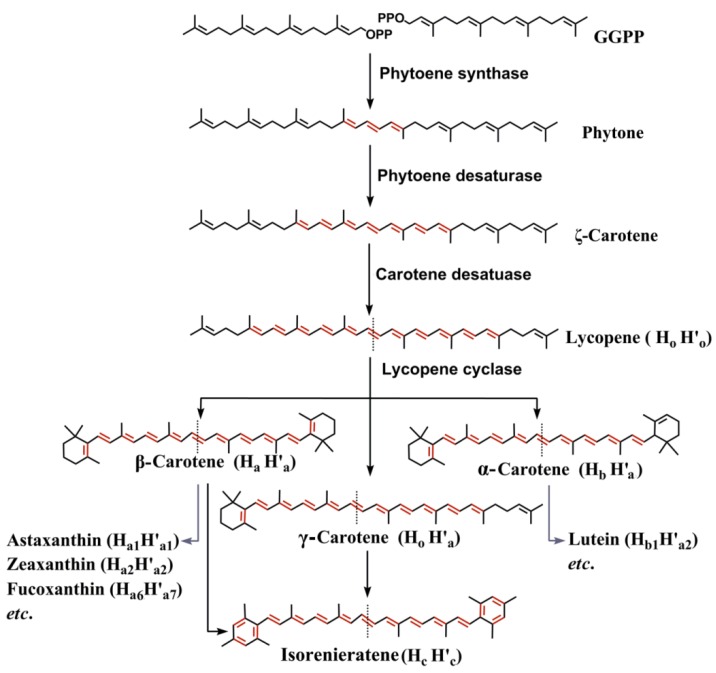
Synthesis pathway of phytoene-based C40 carotenoid backbones. Most C40 marine carotenoids are modified from the backbones of α/β/γ-carotenes or isorenieratene. Carotenoid structures are presented with two symmetric or asymmetric halves (Figure 2), for example, lycopene is shown as H_o_H'_o_ in this review. Conjugated double bonds are shown in red.

Biosynthetic routes to carotenoids begin with the basic building blocks isopentenyl diphosphate (IPP) and its isomer dimethylallyl diphosphate (DMAPP), although carotenoids are very diverse in chemical structure. Two distinct pathways, the 2-*C*-methyl-d-erythritol 4-phospahte (MEP) pathway and the mevalonic acid (MVA) pathway, are responsible for the synthesis of IPP and DMAPP. These two pathways have been reviewed in detail elsewhere [19,20,21,22]. IPP and DMAPP are head-to-tail condensed to generate farnesyl diphosphate (FPP) and geranylgeranyl diphosphate (GGPP) by isoprenyl diphosphate synthases (e.g., IspA of *Escherichia* *coli* and CrtE of *Pantoea* *agglomerans*) [23,24]. As shown in Figure 1, FPP and GGPP are further head-to-head condensed to produce symmetric hydrosqualene (C30) and phytoene (C40), which are dehydrogenated in a stepwise manner by desaturating enzymes representing an important branch point for pathway diversification [25,26].

Enzymes involved in the biosynthesis of carotenoids have been mainly investigated in carotenogenic cyanobacteria and land plants [27,28]. They are mostly associated with cytoplasmic and organelle membranes where the hydrophobic substrates of carotenogenic enzymes are located [29]. So far, very few crystal structures of carotenogenic enzymes have been elucidated because of their association with the membranes [30,31]. More than 95% of carotenoids have been characterized in nature to be phytoene-based [32], which will be extensively discussed in this review.

**Figure 2:**
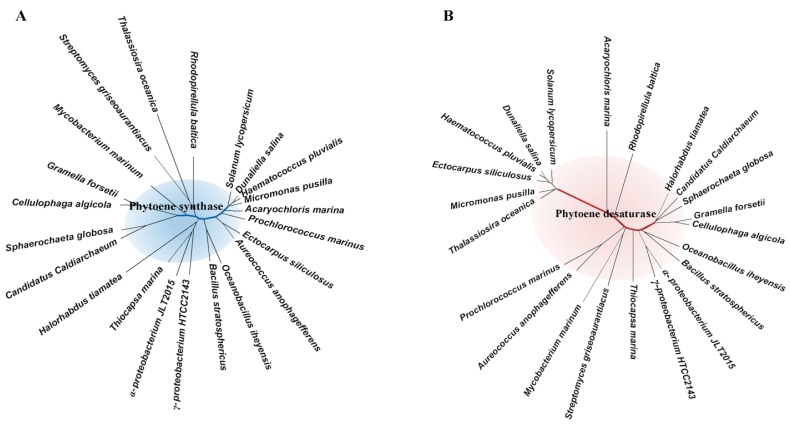
Phylogenic trees of (**A**) phytoene synthases and (**B**) phytoene desaturases. Trees were built using MEGA6.0 software by Neighbor-Joining method [40]. Protein sequences were obtained from National Center for Biotechnology Information (NCBI).

Phytoene synthase is positioned early in the carotenoid synthesis pathway and is responsible as a pathway gatekeeper to discriminate GGPP substrate from enormous isoprenyl diphosphates [29]. Phylogenic analysis of 20 phytoene synthases from marine organisms supports the endosymbiotic theory that plastids evolve from a cyanobacterium, which is engulfed and retained by a unicellular protist [33,34]. Cyanobacteria* Acaryochloris* *marina* and *Prochlorococcus* *marinus* are clustered with green algae and land plant tomato (Figure 2A). However, phytoene synthases still display a significant diversification by evolution. A consensus position of 24.5% (identity of 0.5%) is remained among phytoene synthases from marine algae, bacteria, Achaea and land plants. There is only a similarity of 31.9% even between the two proteobacteria phyla α-proteobacteria and γ-proteobacteria.

The photochemical properties of a carotenoid depend on the size of the chromophore formed by conjugated double bonds, and a C40 backbone can accumulate up to 15 conjugated double bonds [35]. Thus, six sequential desaturation steps are required to dehydrogenate colorless phytoene, which has three conjugated double bonds in the center [26]. Lycopene containing a chromophore with eleven conjugated double bonds is the direct precursor of α/β/γ-carotenes or isorenieratene, the phytoene-based C40 carotenoid backbone (Figure 1). In general, oxygenic phototrophs require three enzymes, phytoene desaturase, ζ-carotene desaturase and *cis*-carotene isomerase to generate lycopene [6]. However, most bacterial phytoene desaturases are able to catalyze all three reactions [30]. There are also some organisms that disobey this general rule. Primitive cyanobacteirum *Gloeobacter* *violaceus* PCC 7421 uses bacterial type phytoene desaturase, and no homolog of ζ-carotene desaturase or *cis*-carotene isomerase is found in its genome [36,37]. Among anoxygenic phototrophs, green sulfur bacteria use three enzymes to catalyze desaturation, whereas purple bacteria, green filamentous bacteria, and heliobacteria use only one enzyme [38,39]. Phytoene desaturases also exhibit significant diversities among different organisms (Figure 2B). Just as phytoene synthase, green algae are clustered with tomato but they are distinguished from cyanobacteria. There is just a similarity of 23.2% among 21 proteins. It is suggested that phytoene desaturase exhibits a much faster evolution from the ancestral blueprint and higher diversities among species than phytoene synthases, which may correspond to promiscuous activities of phytoene desaturase.

### 2.2. Diversity of Marine Carotenoids 

**Figure 3:**
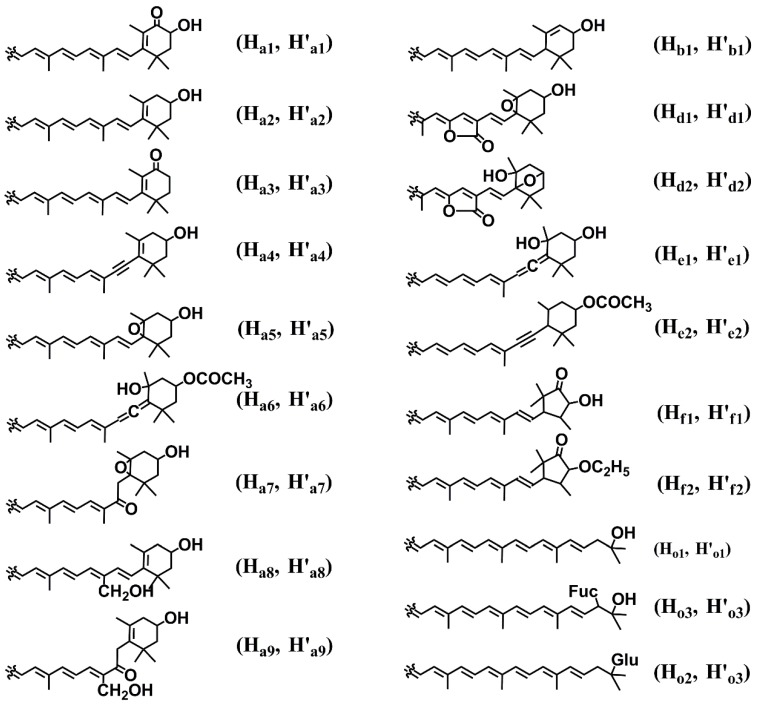
Diverse modifications of carotenoids. The structure shows the modification of a half carotenoid backbone. The stereo configurations are not shown in the structures. Glycosyl moieties of fucose and glucose are represented by Fuc and Glu, respectively.

Carotenogenic organisms in ocean are algae and bacteria, which possess all the genes for *de* *novo* synthesis of carotenoids [2,3,4]. Unicellular microalge *Dunaliella* *salina* and *Dunaliella bardawil* are rich in the orange pigment β-carotene (H_a_H'_a_, Figure 1) [41,42]. Two rings of β-carotene are often oxidized to form astaxanthin (H_a1_H'_a1_, Figure 3) in some microalgae by β-carotene hydroxylase and ketolase [43], which can individually catalyze the modification of β-carotene to generate zeaxanthin (H_a2_H'_a2_, Figure 3) in *Spriulina* *platensis* and *Spriulina* *maxima* [44], and canthaxanthin (H_a3_H'_a3_, Figure 3) in *Haematococcus* *pluvialis*, *Clorella* *vulgaris* and *Colastrella* *striolata* [44,45,46]. The modifications can just occur in one ring to generate asymmetric intermediates such as β-cryptoxanthin (H_a_H'_a2_, Figure 3) and echinenone (H_a_H'_a3_, Figure 3). Chlorophyta *Scenedesmus* *almeriensis* and *Muriellopsis* *sp*. accumulate a large amount of lutein (H_b1_H'_a2_, Figure 3), which is derived from α-carotene (H_b_H'_a_, Figure 1) [47]. Cryptophyta also synthesize α-carotene as well as acetylenic derivatives crocoxanthin (H_b_H'_a4_, Figure 3) and monadoxanthin (H_b1_H'_a4_, Figure 3) [28]. Acetylenic groups are also found in β-carotene derivatives alloxanthin (H_a4_H'_a4_, Figure 3) in Cryptophyta [48], and diatoxanthin (H_a2_H'_a4_, Figure 3) and epoxy oxidized diadinoxanthin (H_a4_H'_a5_, Figure 3) in Heterokontophyta, Haptophyta, Dinophyta, and Euglenophyta [28,49,50]. The unique acetylenic carotenoids are only found in algae. In brown algae and diatoms, acetylated and unique allenic modifications produce dinoxanthin (H'_a5_H_a6_, Figure 3) and chain-oxidized fucoxanthin (H_a6_H'_a7_, Figure 3) [2,51]. Some Chlorophyta species modify the methyl group of lutein to generate loroxanthin (H_b1_H'_a8_, Figure 3) in *Scenedesmus* *obliquus* and *Chlorella* *vulgaris* [52], and siphonaxanthin (H_b1_H'_a9_, Figure 3) in *Codium* *fragile* [53]. Aromatic isorenieratene (H_c_H'_c_, Figure 1) is a usual biomarker compound, which is synthesized from β-carotene in actinobacteria or γ-carotene (H'_o_H_a_, Figure 1) in green and purple sulfur bacteria [54,55]. γ-Carotene can also be converted to chlorobactene (H_c_H'_o_, Figure 1) and OH-chlorobactene (H_c_H'_o1_, Figure 3). Glycoside modifications generate OH-chlorobactene glucoside (H_c_H'_o2_, Figure 3) in green sulfur bacteria and myxol 2'-fucoside (H_o3_H'_a2_, Figure 3) in Cyanophyta [54,56]. Dinophyta can synthesize C37-skeletal carotenoids such as peridinin (H_d1_H'_a6_, Figure 3) [57]. Animals do not have pathways for *de* *novo* synthesis of carotenoids, but they obtain carotenoids from food and further modify carotenoids by oxidation, reduction, translocation of double bonds, cleavage of double bonds,* etc.* Peridinin-originated carotenoids such as peridininol (H_e1_H'_d1_, Figure 3) and cyclopyrrhoxanthin (H_e2_H'_d2_, Figure 3) have been isolated from bivalves *Crassostrea* *gigas*, *Paphia* *amabillis,* and *Corbicula* *japonica* [58,59,60]. Two unique nor-carotenoids, 2-nor-astaxanthin (H_f1_H'_a1_, Figure 3) and actinoerythrin (H_f2_H'_f2_, Figure 3), have been found in sea anemones *Actinia* *equine* and *Tealia* *feline* [61]. The carotenoid diversity in marine animals has been well summarized in detail elsewhere [61]. It is also worthy to note that some carotenoids are present in different stereo configurations among organisms (not covered in this review), which also greatly contributes to the diversification of carotenoids.

### 2.3. Synthesis of Some Important Marine Carotenoids and Enzymes

**Figure 4:**
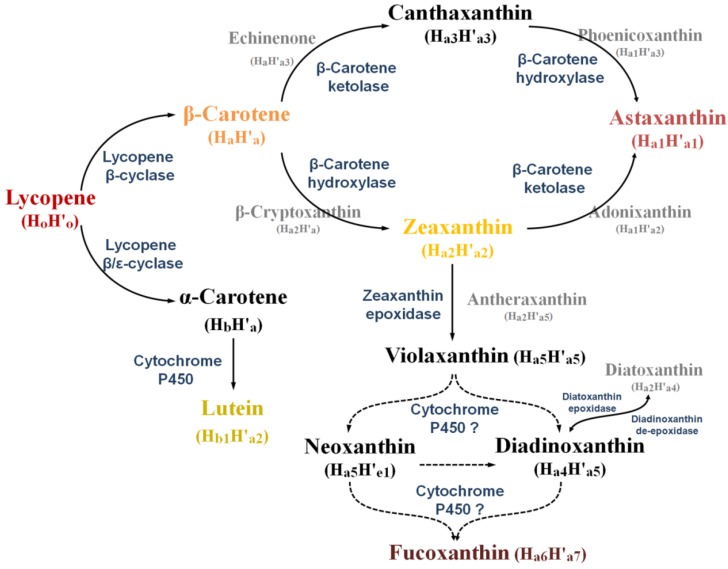
Synthetic pathway of astaxanthin, lutein, and fucoxanthin from lycopene. Arrows indicate each catalysis reaction, and enzymes are shown in blue. Dashed arrows indicate hypothesized reactions. Reaction intermediates are shown in gray.

β-Carotene as well as xanthophylls astaxanthin, zeaxanthin, lutein, and fucoxanthin are some representative marine carotenoids due to their abundance in marine organisms and their inherent antioxidant properties. β-Carotene is synthesized from the cyclization of lycopene, a key step in generating carotenoid diversity by lycopene cyclases, which can also lead to α/γ-carotene formation (Figure 1). The β-cyclase catalyzes the symmetrical formation of two identical β-ionone rings of β-carotene. On the other hand, α-carotene contains two different ring structures (ε and β) formed by the action of additional ε-cyclase with β-cyclase. Four distinct families of lycopene cyclases, CrtY-type β-cyclases in proteobacteria, CrtL β/ε-cyclases in some cyanobacteria, the heterodimeric cyclases in some Gram-positive bacteria and FixC dehydrogenase superfamily lycopene cyclases in *Chlorobium tepidum* and *Synechococcus sp*. PCC 7002, have been identified to date [62]. Further decorations occur via a variety of ketolation (oxidation), hydroxylation (Figure 4), which are the major causes for the diversity among carotenoids [29]. β-Carotene ketolase (CrtW or CrtO) adds the keto groups at the 4,4′-position of the ring and β-carotene hydroxylase (CrtZ) adds the hydroxyl group at the 3,3′-position [63]. Both enzymes are responsible for the formation of astaxanthin via zeaxanthin or canthaxanthin routes in some cyanobacteria and algae (Figure 3). Lutein formation is ascribed to the hydroxylation of α-carotene by cytochrome P450 enzymes in *Arabidopsis* *thaliana* [64], while the pathway and enzymes remain to be elucidated from marine organisms. Fucoxanthin with a unique allenic and epoxide structure is derived from zeaxanthin in brown seaweeds, diatoms and dinoflagellates. Genome analysis indicates that zeaxanthin epoxidases epoxidize zeaxanthin to form antheraxanthin (H_a2_H'_a5_, Figure 3) and violaxanthin (H_a5_H'_a5_, Figure 3) [65]. Two possible routes have been proposed for the synthesis of fucoxanthin from violaxanthin via neoxanthin (H_e1_H'_a5_, Figure 3) or diadinoxanthin [66]. Very recently, a cytochrome P450-type carotene hydroxylase (PuCHY1) has been isolated from red alga *Porphyra* *umbilicalis*. The compensatory expression of PuCHY1 results in the formation of violaxanthin, neoxanthin, and lutein in *A*. *thaliana* by the β/ε-hydroxylation activities [67]. Some of the carotenogenic enzymes characterized from marine organisms have been summarized in the literature [28]. 

## 3. Technology Developments for Production of Carotenoids

Over the decades, many researches have been done for the production of carotenoids. Carotenogenic pathways have been identified and manipulated in several organisms, and advances in metabolic engineering and synthetic biology have resulted in significant improved production of carotenoids including astaxanthin, zeaxanthin, and lutein.

### 3.1. Easy Colorimetric Screening of Production of Carotenoids

**Figure 5:**
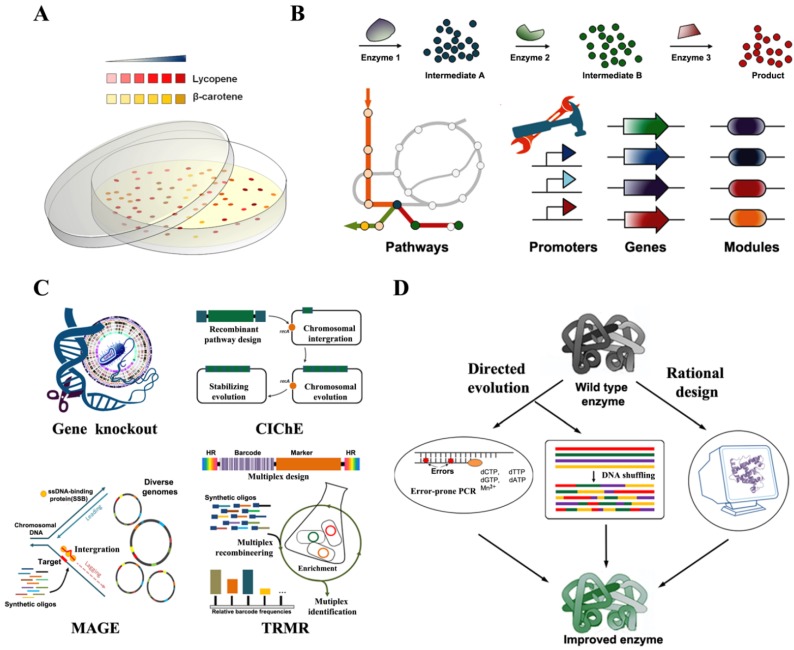
Technologies and efforts to improve carotenoid production. (**A**) Colorimetric screening of desired producer; (**B**) Pathway engineering approaches for production improvement; (**C**) Genetic modifications for host strain development; (**D**) Protein engineering for enzyme and pathway improvement.

Carotenoids contain chromophores absorbing visible light and appear as being yellow (e.g., β-carotene) to red (e.g., lycopene), which benefits carotenogenic gene mining and engineering upon carotenoid synthesis pathway. To date, many carotenoid biosynthetic genes have been cloned from plants, bacteria, and fungi based on their abilities to render different colors to the host [68,69,70]. This merit has been vigorously implemented for random mutagenesis, directed evolution, and proof-of-principle experiments in synthetic biology. Moreover, cellular carotenoids can be easily extracted into an organic solvent and differentiated in a sensitivity of submilligrams per liter with a linear correlation between carotenoid contents and color intensity [71,72]. This provides an easy and high-throughput way to evaluate the performance of newly built synthetic circuits or methodologies for improved biosynthesis of carotenoid (Figure 5A).

### 3.2. Pathway Engineering for Production of Carotenoids

Carotenoid biosynthesis emerges from the central isoprenoid pathway, either the MEP pathway or the MVA pathway, existing in all organisms [19,22]. The expression of carotenogenic genes can yield carotenoids of interest in a heterologous organism [16,73,74,75]. The early attempts led to the production of lycopene, β-carotene, and astaxanthin in *Saccharomyces* *cerevisiae* and *Candida* *utilis* by the expression of carotenogenic enzymes from *Pantoea* *ananatis* [74,76]. *Corynebacterium glutamicum* is a native producer of decaprenoxanthin and its glucosides, and it has been engineered to synthesize C50 carotenoids C.P.450 and sarcinaxanthin [77]. To date, there have been many exemplary illuminations to achieve high carotenoid titers from non-native producers. Carotenogenic enzymes from different sources exhibit different capacities in carotenoid biosynthesis. A two-fold higher lycopene production is obtained in *E*. *coli* by the expression of carotenogenic enzymes from *P*. *agglomerans* (27 mg/L) than from *P*. *ananatis* (12 mg/L) [78]. Metabolic engineering approaches allow the assembly of genes from different organisms for production purposes or for building new carotenoids [32,79,80]. β-Carotene production has been improved by hybrid expression of carotenogenic genes from *P*. *agglomeras* and *P*. *ananatis* in *E*. *coli* [81]. In another example, expression of β-end ketolase from *Agrobacterium* *aurantiacum* extends the zeaxanthin β-d-diglucoside pathway from *P*. *ananatis*, and synthesizes novel astaxanthin β-d-diglucoside and adonixanthin β-d-diglucoside [29]. Generally, a sufficient precursor supply is a prerequisite for high-yield production of carotenoids. Overexpression of the rate-limiting enzymes 1-deoxy-d-xylulose-5-phosphate synthase and reductoisomerase led to a 3.6-fold increase in lycopene production in *E*. *coli* when compared with the native MEP pathway for IPP and DMAPP supply [71]. Overexpression of the rate-limiting enzyme 3-hdroxy-3-methyl-glutaryl-coenzyme A (HNG-CoA) reductase of the MVA pathway from *Xanthophyllomyces* *dendrorhous* significantly increased β-carotene production in *S*. *cerevisiae* [82]. A great effort in metabolic engineering of the central carotenoid building block pathway is the introduction of a hybrid MVA pathway of *Streptococcus* *pneumonia* and *Enterococcus* *faecalis* into *E*. *coli*, which enables the recombinant host to produce 465 mg/L of β-carotene [83]. With more available genetic tools, microbial organisms such as *Pseudomonas putida* and *Bacillus* *subtilis* have also been developed as platform hosts for carotenoid production [84,85].

Carotenoids synthesis involves multiple enzymes [2]. The expression level of all the components of a multigene circuit should be orchestrated to optimize metabolic flux and to gain a high yield (Figure 5B) [86]. A random approach is screening of the best orchestra from numerous combinatorial assemblies of required genes and control elements. BioBrick™ paradigm is capable of rapidly assembling a biosynthetic pathway in a variety of gene orders from different promoters in plasmids with different copy numbers [87]. It is possible to build a hybrid carotenoid pathway wherein each enzyme possesses a right turnover number, however, BioBrick™ assembly is still not in a high throughput to create vast combinatorial expression constructs for the best combination of carotenogenic genes. Recently, several advanced assembly methods using homologous recombination, such as sequence and ligation-independent cloning (SLIC), Gibson DNA assembly and reiterative recombination, have been applied to construct multigene circuits [88,89,90]. These advances promise to randomize all genetic components, including genes, promoters, ribosome binding sites, and other control modules to build a large number of individual genetic circuits for screening purposes. A so-called “randomized BioBrick assembly” approach has been applied to the optimization of the lycopene synthesis pathway wherein the expression construct was designed to independently express each enzyme from its own promoter, which resulted in an increase by 30% in lycopene production [91]. A longer and more complicated pathway can be modularized into subsets, which contain pathway enzymes with similar turnover numbers. Modulating these subsets would be more convenient and efficient than regulating all components of the entire pathway for improved production [92]. By using this multivariate modular metabolic engineering (MMME) approach, recent work achieved a 15,000-fold increase in production of taxadiene, a precursor of the anti-cancer drug taxol [93]. There are also a variety of promising approaches, such as tunable promoters, tunable intergenic regions, and ribosome binding site design, which can be applied to fine tuning the expression of modules [94,95,96]. In the other approaches, a multi-genic operon is transcribed into a single polycistronic mRNA, and then the large transcript can be spliced to small monocistronic transcripts through post-transcriptional RNA processing such as ribozyme cleavage and clustered regularly interspaced short palindromic repeats (CRISPR) editing. Thus, the stability of the monocistronic transcripts can be independently modulated to differentiate the expression level of each enzyme even in a multi-gene operon. These RNA processing tools have been developed as insulating elements between operonic genes to reduce the context dependence of the genes in a polycistronic transcription unit [97]. The diffusion of pathway intermediates can decrease the effective concentrations of intermediates for following enzyme reactions and some intermediates may serve for competing pathways. By learning from Mother Nature, synthetic biologists spatially organize enzymes of the MVA pathway by protein scaffolds in *E*. *coli* to minimize diffusion limitation and achieve a 77-fold increase in mevalonic acid production [98]. The propanediol utilization machinery of *Citrobacter* *freundii* has been heterologously recasted in *E*. *coli* [99]. Some intermediates of carotenoid synthesis such as isoprenyl diphosphates are toxic when they accumulate over the concentration threshold [100]. To avoid the accumulation of toxic intermediates, genetic sensors can potentially be coupled with gene expression cassettes to regulate the intermediate flux in a dynamic manner. The native *E*. *coli* promoters that respond to the toxic FPP have been successfully used to dynamically regulate the amorphadiene synthesis pathway and improve the production by two-fold over common inducible promoters and constitutive promoters [101]. The Ntr regulon has been engineered to control lycopene synthesis in response to glycolytic flux dynamics, resulting in an 18-fold increase in lycopene production [102].

### 3.3. Genome Engineering for Strain Development

For the most efficient carotenoid production, the biological system of the host organism also needs to be optimized, by, for example, redirecting cellular carbon flux to the carotenoid synthesis pathway. The *de* *novo* synthesis of carotenoids is initiated from acetyl-coA by the MVA pathway or glycolytic metabolites pyruvate and glycraldehyde-3-phosphate (G3P) by the MEP pathway. The direct efforts are focused on the modification of associated genes to these pathways. Deletion of pyruvate kinases PykFA can balance the availability of pyruvate and G3P for the MEP pathway, and increase lycopene production by 2.8-fold in *E*. *coli* [103]. The deletion of glucose-6-phospahte (G6P) dehydrogenase Zwf, which branches G6P to pentose phosphate pathway results in an increase by 30% in lycopene production [104]. Deletion of carbohydrate phosphotransferase system yields a seven-fold increase in lycopene production in another study [105]. Replacement of native promoters of the rate-limiting genes of the MEP pathway with the T5 promoter has been carried out for enhancement of the targeted pathway flux, which results in a 4.5-fold increase in β-carotene production [106].

A heterologous pathway is not just an independent entity. It communicates with the native cellular metabolism and is therefore governed by the global regulation of the host organisms. Adaptive laboratory evolution is a traditional route for strain engineering to achieve desirable industrially relevant phenotypes. Owing to the antioxidant properties of carotenoids, adaptive evolution has been successfully applied to an engineered *S*. *cerevisiae* with periodic hydrogen peroxide shocking, resulting in a three-fold increasee of β-carotene production. Subsequent transcriptome analysis indicates that some genes related with lipid biosynthesis and MVA pathways are up-regulated in the adopted strains [107]. It also suggests that carotenoid production can be improved by modifications (knock-out or overexpression) of distant genes, which are responsible for the overall regulation of the metabolic network or the physiological fitness of the host (Figure 5C). In a genome-wide screening of yeast deletion collection, 24 deletions exhibit significant higher carotenoid levels than the wild type. The triple deletion of *ROX1*, *YJL064W*, and *YJL062W* shows an almost four-fold increase in total carotenoid production [108]. Gene deletions of *hnr*, *yjfP*, and *yjiD* related to the improvement of lycopene production have been identified from a global transposon *E*. *coli* mutant library [109]. Other gene deletions such as* gdhA*, *cyoA*, *ppc*, *gpmA*, *gpmB*, *eno*, *glyA*, aceE, *talB*, and *fdhF* have been *in* *silico* identified using a stoichiometric model [110]. The triple mutation of *gdhA*, *aceE* and *fdhF* was validated to increase lycopene production by nearly 40% in *E*. *coli* over the engineered parental strain. A similar set of gene deletions *dhA*, *cyoA*, *gpmA*, *gpmB*, *icdA*, and *eno* have been also *in silico* identified using different metabolic network models [111]. Overexpression of some genes encoding global regulatory proteins AppY, Crl, RpoS, and ElbAB, oxidoreductases TorC, YdgK, and YeiA, and hypothetical proteins YedR and YhbL, result in a significant increase in lycopene production in *E*. *coli* [112]. With a profound understanding of the landscape of genome manipulation, all these knocked-out and overexpressed alleles have been combined and optimized to generate high-fitness host strains for lycopene production [113,114]. ATP and NADPH are also important cofactors for the production of carotenoids. Using engineering ATP synthesis, pentose phosphate and TCA modules, recent work has shown the highest β-carotene production of 2.1 g/L by a fed-batch fermentation process in *E*. *coli* [115]. The advances in synthetic biology greatly boost genome manipulation on a large scale. Multiplex automated genome engineering (MAGE) simultaneously targets many locations on the chromosome for modification in a single cell or across a population of cells by directing ssDNA to the lagging strand of the replication fork during DNA replication [116]. The modifications can cover gene inactivation, expression regulations, and so on. Aforementioned twenty genes related to lycopene production have been targeted to tune their expression using a complex pool of synthetic DNAs, and lycopene production is increased more than five-fold. A complementary method called trackable multiplex recombineering (TRMR) has been developed to simultaneously map genome modifications that affect a trait of interest, which combines parallel DNA synthesis, recombineering and molecular barcode technology to enable rapid modification of all *E*. *coli* genes in an *a priori* knowledge-independent way [117].

Metabolic engineering for the production of valuable compounds often heavily relies on plasmid-based expression of the synthesis pathway in a heterologous host. Although plasmids are easily manipulated and allow strong expression of targeted enzymes, the plasmid-based systems suffer from genetic instability such as plasmid loss, an additional antibiotic cost, and a potential risk of antibiotic marker spreading to other organisms [118]. Accordingly, chromosomal integration of the production pathway promises the host to achieve stable overproduction of the desirable chemicals including carotenoids. By λ-Red homologous recombination, plasmid-free engineered *E*. *coli* strain has been developed to produce lycopene and astaxanthin [119]. The expression cassettes can be integrated into different loci to increase the number of gene copies. P1 transduction usually plays a role in transfering the different alleles between host strains. Recently, an intelligent strategy called chemically inducible chromosomal evolution (ClChE) has been developed to reduce the daunting repeated one-at-a-time tasks in the chromosomal integration of target genes [88]. ClChE allows the host to acquire a high gene copy (up to 40 copies) expression of integrated pathways with increasing concentration of selective chemicals, and the increased copy number is stabilized by the removal of the *recA* gene. With this approach, lycopene production has been increased by 60% from single copy integrated strain. The ClChE strategy has been further modified to eliminate antibiotic marker for environmental safety and health issue after the evolution of the recombinant host strain [120].

### 3.4. Protein Engineering for Improvement of Carotenoid Production Enzymes

Pathway engineering for efficient production of desired chemicals is often challenged by limitations associated with the pathway enzymes themselves, such as low turnover numbers and promiscuities generating unwanted by-products [121]. Protein engineering provides a powerful solution to improve specific activity and substrate specificity of enzymes, and even to create new activity. Methods of protein engineering include directed evolution and computer-assisted rational design (Figure 5D) [122,123]. Directed evolution is an iterative process that imitates Darwinian evolution in the laboratory to select or screen a desired phenotype from mutagenesis. Typically, error-prone polymerase chain reaction (PCR) is used to generate mutant libraries, and DNA shuffling is carried out to recombine existing mutations. It can be performed in a blind manner with limited information on target enzymes, such as structures and reaction mechanisms, but it relies on an effective screening strategy. It is practical for the evolution of carotenogenic enzymes due to the innate traits of carotenoid pigments. Six mutants ((H96L, R203W, A205V, A208V, F213L and A215T) have been isolated to improve the catalytic activity of β-carotene ketolase from *Sphingomonas* *sp*. [124]. Three mutations (L175M, M99V, and M99I) of ketolase from* Paracoccus* *sp*. result in the improvement if its specificity of to synthesize astaxanthin [125]. *Staphylococcus* *aureus* dehydrosqualene (C30) synthase has evolved to synthesize lycopene by mutation F26L or F26S [126]. DNA shuffling of phytoene desaturases from *P*. *agglomerans* and *P*. *ananatis* results in the isolation of a variant favoring the production of fully conjugated tetradehydrolycopene [127]. Rational design of proteins is based on the *in* *silico* simulation and the prediction using *a priori* enzyme information, which greatly liberates biologists from onerous screening task. This strategy requires adequate information to predict specific targeted amino acid mutations, which can confer desired enzyme traits [128]. Unfortunately, the limited information on carotenogenic enzymes leads to few achievements using such a method.

As aforementioned, carotenoids are derived from the central isoprenoid pathway, which is also employed to synthesize several essential and secondary metabolites in nature. The carotenoid-based colorimetric screening has been developed for evolution of other isoprenoid pathway enzymes. Mutations of GGPP synthase are hypothesized to affect the binding efficiency of the magnesium ions needed for substrate anchoring and improve its catalysis. An error-prone PCR library of *Tsuga* *canadensis* GPPS has been screened using the lycopene synthesis pathway as a colorimetric reporter. The GPPS variant (S239C and G295D) is created to increase levopimaradiene production with a 1.7-fold increase over the wild type in *E*. *coli* [129]. Augmentation of one pathway can tamper with other pathways, which utilize the same substrate in one organism. Based on this fact, mutagenesis libraries of terpene synthases have been screened by depigmentation of colonies due to the competition between terpene synthases and carotenoid synthases for isoprenyl diphosphates, since the weakened carotenoid color intensity indicates an improvement of terpene synthase activity [130].

### 3.5. Development of Microalgae for Carotenoid Production

Algae are a diverse group of aquatic, photosynthetic organisms, generally categorized as macroalgae (*i*.*e*., seaweed) and unicellular microalgae. Microalgae have recently garnered interest for production of valuable chemicals including carotenoids [41,131], because they are generally regarded as safe (GRAS) for human consumption and possess the renewable-energy capturing ability of photosynthesis. Moreover, these organisms can be used for genetic manipulation and high-throughput analysis [132]. Some microalgae are also native carotenoid producers (*i*.*e*., *D.* *salina* for β-carotene and *H*. *pluvialis* for astaxanthin). The carotenoid production from microalgae is closely related to culture conditions such as illumination, pH, temperature, nitrogen availability and source, salinity, the oxidant substances, and growth rate [12,133,134]. *D*. *salina* is a model species of green microalgae which is widely cultivated outdoors for β-carotene production [131]. A systematic evaluation has been done to decipher the relationship between abiotic stresses (Nitrate concentration, salinity and light quality) and lutein synthesis in *D.* *salina* [135]. The abiotic stresses can also be applied to adaptive evolution of microalgae [136], in a similar manner to strain evolution in yeast for β-carotene production [107]. The freshwater microalga *Chlamydomonas* *reinhardtii* is the first and the best studied transformed Chlorophyte, and the nuclear genetic manipulation is easy and well established. It has been engineered with β-carotene ketolase from *H*. *pluvialis* to synthesize ketolutein (H_b1_H'_a1_, Figure 3) and adonixanthin (H_a1_H'_a2_, Figure 3) [137]. It is possible to produce diverse valuable carotenoids from marine microalgae with the development of more available genetic tools and technologies.

## 4. Opportunities and Challenges

The vast and mysterious ocean breeds diverse marine lives and provides unexhausted foodstuffs, nutriment, and drugs for humans. Diverse carotenoids are found from marine species and show broad utilities as colorant fragrance cosmetics and pharmaceuticals. The synthetic pathway of several carotenoids has been illuminated from marine species, which could benefit engineering processes in several host organisms for the production of carotenoids such as β-carotene, astaxanthin, and lutein. On the other hand, carotenoids such as β-carotene often undergo a series of modifications in the miraculous marine ecosphere. And indeed, several novel carotenoids have been isolated during the exploration of the marine ecosphere, while their pharmaceutical potentials remain to be examined due to the limited amount of extracts. Metabolic engineering and synthetic biology allow the assembly of such a chimeric pathway in a tractable organism for the mass production of rare carotenoids and also exhibit the potential to extend the catalogs of carotenoids to non-natural carotenoids, which could accelerate the exploration of novel carotenoids. It is noted that decoded carotenoid pathways and enzymes are still limited to a few marine organisms, although the J. Craig Venter Institute with worldwide collaboration had sequenced and annotated the genomes of 177 marine microbes up until 2010. However, we believe that the developed and developing technologies will allow us to search for novel marine carotenoid pathways in the future.
